# Ginsenoside Rh2 mitigates doxorubicin‐induced cardiotoxicity by inhibiting apoptotic and inflammatory damage and weakening pathological remodelling in breast cancer‐bearing mice

**DOI:** 10.1111/cpr.13246

**Published:** 2022-05-09

**Authors:** Jingang Hou, Yeejin Yun, Changhao Cui, Sunchang Kim

**Affiliations:** ^1^ Intelligent Synthetic Biology Center Daejeon Republic of Korea; ^2^ Department of Biological Sciences Korea Advanced Institute of Science and Technology Daejeon South Korea; ^3^ Research and Development Team 4, Sempio Foods Company Cheongju South Korea

## Abstract

**Objectives:**

There are presently a few viable ways to reduce cardiotoxicity of doxorubicin (Dox). The combination of chemotherapy agents with natural compounds delivers greater efficacy and reduces adverse effects in recent researches for cancer treatment. Here, we examined the potential effect of ginsenoside Rh2 on a Dox‐based regimen in chemotherapy treatment.

**Materials and Methods:**

Human breast tumour (MDA‐MB‐231) xenograft nude mice, human cardiac ventricle fibroblasts, and human umbilical vein endothelial cells (HUVEC) were employed in the present study. Histology, immunohistochemistry, immunofluorescence, western blot, antibody array, and RNA‐sequencing analyses were utilized to assess the protective effect of Rh2 on cardiotoxicity induced by Dox and the underlying mechanisms.

**Results:**

Rh2‐reduced cardiotoxicity by inhibiting the cardiac histopathological changes, apoptosis and necrosis, and consequent inflammation. Pathological remodelling was attenuated by reducing fibroblast to myofibroblast transition (FMT) and endothelial–mesenchymal transition (EndMT) in hearts. RNA‐sequencing analysis showed that Dox treatment predominantly targets cell cycle and attachment of microtubules and boosted tumour necrosis, chemokine and interferon‐gamma production, response to cytokine and chemokine, and T cell activation, whereas Rh2 regulated these effects. Intriguingly, Rh2 also attenuated fibrosis via promoting senescence in myofibroblasts and reversing established myofibroblast differentiation in EndMT.

**Conclusions:**

Rh2 regulates multiple pathways in the Dox‐provoked heart, proposing a potential candidate for cancer supplement and therapy‐associated cardiotoxicity.

## Graphical abstract

Doxorubicin is extensively reported to induce severe cardiotoxicity in clinical applications. Our work proposed a natural herbal compound, ginsenoside Rh2, as a potential candidate for attenuating this side effect. Rh2 significantly inhibited cardiac apoptosis and necrosis, inflammation, and pathological remodelling in Dox‐challenged hearts.

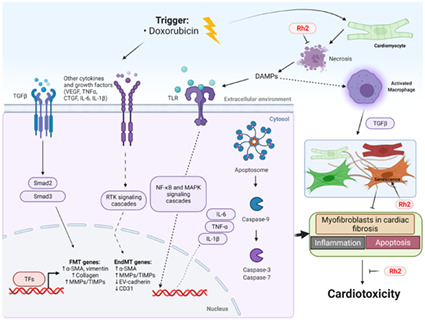

## 1 INTRODUCTION

In the United States, approximately 1,898,160 cancer cases are diagnosed each year. Of these, over 280,000 are breast cancers accounting for 30% of female cancers, with estimated deaths accounting for 15% in all‐female incident deaths.^1^ Thus, a large portion of the population is impacted by the disease, making it a critical public health issue. Consequently, it has attracted numerous research interests. The options of treatment regimens for breast cancer depend on various criteria, namely the stage and subtype of cancer, oestrogen and hormone receptor status (positive or negative), and the overall health condition (other diseases or cancer). Given this existing complexity, it's challenging to propose a universally approved treatment. The right choice of therapy should take into account the benefits over risks. Currently, main therapeutic drugs approved by Food and Drug Administration (FDA) to treat or prevent breast cancers include Tamoxifen Citrate, Abemaciclib, Atezolizumab, Cyclophosphamide, Docetaxel, Doxorubicin Hydrochloride, Epirubicin Hydrochloride, 5‐Fluorouracil, Gemcitabine Hydrochloride, Lapatinib Ditosylate, Methotrexate, Ribociclib, Tucatinib, etc.^2^, ^3^, ^4^ Anthracyclines such as doxorubicin are a class of most principle chemotherapeutic agents among those mentioned above, which is widely used as adjuvant therapy for the initial treatment of breast cancer and the treatment of incurable metastasis and occurrence of relapses in patients with breast cancer.^5^ However, doxorubicin provokes copious irreversible cardiotoxic side effects, which even increases the risk of heart failure.^6^ The multitudinous doxorubicin cardiotoxicity gives rise to the major challenge for developing systemic treatment regimens. Although the molecular and genetic mechanism of doxorubicin‐induced cardiotoxicity is not completely understood, the presence of several vended studies is believed to the production of free radicals in both normal and malignant cells,^7^ direct damage to mitochondria induced by reactive oxygen species (ROS),^8^ downregulation of uncoupling proteins,^9^ depleted cardiac stem cells^10^ and impaired DNA synthesis.^11^ Collectively, these actions greatly hinge the clinical usage of doxorubicin.

Considering the multiple and interacted molecular pathways in cancer, there is a big possibility that tumours are simultaneously sensitive to different drugs. This makes it achievable to study the combinations of drugs with less toxicity, higher efficacy, decreased dosages, and potentially antagonizing drug resistance in cancer therapy.^12^ Doxorubicin combined with cyclophosphamide (AC) and the addition of paclitaxel (AC‐T) were identified as highly effective therapies for early breast cancer and metastatic breast cancer with shorter time and fewer visits in American.^13^ However, with lower dosages, these treatments may still be associated with moderate to serious toxicities that decrease the overall quality of life for patients since all the ingredients are consisted of chemotherapy agents. Repurposing drugs have been proposed such as fidarestat,^14^ digoxin,^15^ and metformin^16^ to synergistically prevent breast cancer and concomitantly reduce doxorubicin‐induced cardiotoxicity. These approved therapeutic drugs may offer certain treatment options for cancer patients, however, more trials are required to obtain maximum tolerated doses for oncologic purposes, particularly in the combinations with other agents. Furthermore, Pegylated liposomal doxorubicin represents an improved formulation of doxorubicin, with reduced cardiotoxicity and improved pharmacokinetic profile, but still leads to certain additional new side effects on clinical trials.^16^

Natural compounds that are not originally intended for cancers are reported as chemotherapeutic drug sensitizers and chemopreventive effectors. They significantly increase the antitumor effects, minimize the drug resistance and prevent the adverse effects.^17^ Importantly, natural compounds have diverse bio‐functional modalities in a vast array of conditions, which facilitates their application in many treatment complications. In the present study, we have addressed the synergistic effects of ginsenoside Rh2 on doxorubicin‐treated breast cancer‐bearing mice. We found that Rh2 may significantly enhance the antitumor effects of doxorubicin and reduce cardiotoxicity during the therapy phase.

## 2 MATERIALS AND METHODS

### 2.1 Reagents

Doxorubicin hydrochloride (Dox), Retinoic acid (RA), Xylene, Ethanol, Tween‐20, and Methanol were purchased from Sigma‐Aldrich (St. Louis, MO. USA). Difco™ Skim Milk was purchased from BD Company (Franklin Lakes, NJ, USA). Fetal bovine serum (FBS), Penicillin, streptomycin, were purchased from Gibco‐Invitrogen (Grand Island, NY), Laemmli buffer, polyvinylidene difluoride (PVDF) membranes, nitrocellulose membranes, Proceau solution, Tris‐glycine buffer, Tris‐glycine‐SDS buffer, pre‐stained protein marker were purchased from Bio‐Rad (Hercules, CA, USA). Live and fixed cell nuclear imaging kit, Novex 4% to 12% tris‐glycine gels, Bolt™ 12%, Bis‐Tris gels, iBlotTM Transfer Stack, PVDF were obtained from Thermo Fisher Scientific (Waltham, MA, USA). Masson trichrome (MT) and Sirius Red (SR) staining kits were obtained from Abcam Company (Cambridge, UK). Ginsenoside Rh2 with a purity of more than 98% was prepared with HPLC by our research group.

### 2.2 Mice and study design

We conduct in vivo experiments on 6‐week‐old wild‐type BALB/c female mice. The mice were randomly divided into six different groups: Control, Rh2 (20 mg/kg), Rh2 (30 mg/kg), Rh2 (20 mg/kg) + Dox (2 mg/kg), Rh2 (30 mg/kg) + Dox (2 mg/kg), and Dox (2 mg/kg).

In the 3‐week tumour experiment, the mice were injected subcutaneously (s.c.) with a 9 × 10^6^ stable pool of MDA‐MB‐231 cells at day 0. On day 7, the BALB/c nude mice were received injections of Dox dissolved in 0.9% saline every other day with a cumulative dose of 22 mg/kg body weight. Rh2 dissolved in PBS + Tween 80 was injected every day during the treatment course. Tumour volumes were measured every other day using callipers and calculated with the following formula: volume = width × height × length/2. All animal experiments were approved by the Institutional Animal Care and Use Committee of Korea Research Institute of Bioscience and Biotechnology (Approval #: KRIBB‐AEC‐19277).

### 2.3 Immunohistochemistry

Surgically resected tissues were formalin fixed, paraffin embedded, and sectioned onto the slide.

For H&E staining, coronal sections of the heart, transverse planes of tissue were stained using an H&E kit (Abcam) according to the procedure. The sections were observed and imaged with a Nikon Ti2 U microscope (USA).

For cleaved caspase 3 staining, positive staining was detected using a kit (CST) and the manufacturer's protocol. For heart tissue, the coronal section was used for staining. All TUNEL‐positive cells per 40× microscope field were counted. Sections were counterstained with Methyl Green. Positive staining is represented by a dark brown (DAB) signal.

The slides were deparaffinized using xylene, rehydrated, and antigen‐retrieved in a microwave oven for 30 min. After the inhibition of endogenous peroxidase activity, individual slides were washed with PBST (0.05% Tween) and then incubated in blocking solution (1× gelation, Biotium, USA) for 30 min. The primary antibodies in 1% BSA in PBS were incubated overnight at 4°C. Antibodies against the following proteins were used: cleaved‐caspase 3 (CST, USA), α‐SMA (Abcam, USA), p16, CD206 (Santa Cruz, USA), and p21 (Sigma, USA). The sections were then washed with PBST, incubated with Alexa Fluor secondary antibodies or DAB substrate, and mounted with antifades medium with DAPI (Thermo Fisher Scientific). Sections were imaged with a Nikon Ti2 U microscope (USA).

For Masson trichrome (MT) and Sirius Red (SR) staining, sections were stained according to manufacturer's protocols from Abcam Company. Then stained sections were imaged and quantified.

### 2.4 Cell culture

Heart myoblast cells H9c2 (2‐1) purchased from America Tissue Type Collection (Manassas, VA; catalogue # CRL‐1446) were maintained in high‐glucose Dulbecco's Modified Eagle's Medium (Thermo Fisher Scientific), supplemented with 10% Fetal Bovine Serum and penicillin/streptomycin. Cells were cultured with fresh medium (1% FBS + 1 μM RA) daily for 5 days to obtain differentiated cardiomyocytes. Human ventricular cardiac fibroblasts (HCF) were obtained from Lonza (Basel, Switzerland; catalogue # CC‐2904) and were cultured with FGM™‐3 cardiac fibroblast growth medium‐3 bulletkit™ (Lonza, catalogue # CC‐4526). human umbilical vein endothelial cells (HUVEC) obtained from America Tissue Type Collection (catalogue # CRL‐1730) was cultured with EGM™ Endothelial Cell Growth Medium BulletKit™ (Lonza, catalogue # CC‐3124). The cells were incubated in 75 cm^2^ tissue culture flasks at 37°C in a humidified atmosphere of 5% CO2 atmosphere.

### 2.5 Transfection

HCF cells were plated at a density of 2 × 10^5^ cells using a 6‐well‐plate in a complete culture medium. On the transfection day, cells were transfected using mixtures of 12 μl of highperfect transfection reagent (QIAGEN) and 100 nM of siRNAs of control, p21 and p53 (CST, USA) for 48 h. Then, cells were collected and analysed by the western blotting method.

### 2.6 Cell treatment and assay

Cells were treated with 100 nM of doxorubicin for 7 days followed by exposure of 2.5, 5 and 10 μg/ml ginsenoside Rh2 for extra 7 days. Control cells were treated with DMSO and 10 μg/ml Rh2, respectively, as the same indicated durations. Conditioned medium was obtained by culturing the cells with serum‐free medium for extra 2 days after the treatment endpoints. Then, cells or conditioned media were collected and stored at −80°C for the subsequent experiments.

### 2.7 Biochemical analysis

Heart tissues from mice sacrificed at the indicated endpoint or cells with indicated treatments were lysed with a Minute™ total protein extraction kit for animal cultured cells and tissues obtained from Invent Biotechnologies, Inc. (Plymouth, MN, USA). For tissue extraction: about 20 mg tissue was placed in the pre‐chilled filter cartridge, ground with a plastic rod for 60 times with twisting fore, and continued to grind for 60 times in 200 μl native lysis buffer containing proteinase and phosphatase inhibitors in the filter. After incubation on ice for 5 min, tubes were centrifuged for 2 min at 4°C. The supernatant flow‐through contains native protein extract and is subjected to BCA assay for protein‐content determination (Thermo Fisher Scientific). For cell extraction: cells with indicated treatments were rinsed with cold PBS, and placed with an appropriate volume of lysis buffer. Scraped the cells and pipetted up and down repeatedly. Then, transfer the cell lysate to a pre‐chilled filter cartridge and centrifuge at 14000 × g for 30 seconds. The flow‐through in the collection tube was put in ice and assayed by the BCA method.

Analyses for Creatine Kinase MB (CK‐MB, Catalogue # E‐EL‐R1327) and Troponin T (TnT, EKU11492) from BIOMATIK Corporation (Ontario, Canda) were performed following the manufacturer's protocols.

Detection of TNF‐α, IL‐6, and IL‐1β was obtained using kits from RayBiotech Life, Inc. (Norcross, GA, USA) according to the manufacture's manuals. Signals were detected by the Tecan plate reader (Männedorf, Zürich, Switzerland).

Antibody array kit of apoptosis purchased from R&D Systems (Minneapolis, MN, USA) was used according to the manufacture's manuals. Heart tissues (300 μg) extracted from snap‐frozen samples were used in the present study. Signals were detected with the Odyssey‐LC chemiluminescent imaging system (LI‐COR, Lincoln, NE). Signals were averaged and expressed as described in the figure legend.

### 2.8 Immunoblotting

Total protein content was determined using the BCA assay. Twenty micrograms of total protein were separated by SDS‐polyacrylamide gel electrophoresis was performed using Novex 4% to 12% tris‐glycine gels or Bolt™ 10% or 12%, Bis‐Tris gels, and proteins were transferred to PVDF or NC membranes by semidry transfer using iBlot™ 2 Gel Transfer device (Thermo). Membranes were blocked in Odyssey‐blocking buffer (LI‐COR Biosciences) and incubated overnight at 4°C with primary antibodies. After three times repeated washes with TBST [0.05% Tween 20], blots were incubated with the appropriated IRDye‐conjugated or HRP‐conjugated secondary antibody (LI‐COR Biosciences) and imaged using the LI‐COR Odyssey Fc. Bands were quantified using the Odyssey software (LI‐COR Biosciences). The following antibodies were used in the study: caspase 3, cleaved caspase 3, caspase 7, caspase 9, PARP, p21, p16, vimentin, GAPDH, COL1A, (Cell Signalling Technology), CD31, VE‐cadherin (Santa Cruz Biotechnology), α‐SMA (Abcam), and β‐actin (Sigma).

### 2.9 Immunofluorescence staining

Cells with various indicated treatments were washed twice with PBS, then cells were fixed with 4% paraformaldehyde for 10 min and permeabilized with 0.15% Triton X‐100 in PBS for 15 min at room temperatures. Cells were then blocked with 3% BSA for 30 minutes and incubated with indicated primary antibody against p21, (1:2000, CST, USA) overnight at 4°C, followed by incubation with Alexa fluorescein‐labelled secondary antibodies (1:500, Life Technologies, USA) for 1 h and nuclear stain for 5 min (Life Technologies, USA). Images were captured with a Nikon Ti2 U microscope (USA).

### 2.10 RNA‐seq analysis

Hearts were isolated from mice and flash frozen on dry ice. RNA from hearts pooled from five separate mice was extracted using the RNeasy Mini kit (Qiagen) following the manufacture's manual. RNA sequencing was carried out by Macrogen Inc. (Seoul, Korea). SureSelect XT RNA Direct Reagent kit was used for the construction of sequencing libraries. The libraries were sequenced on an Illumina sHiSeq2500 (Illumina, San Diego, CA, USA) instrument to generate 101 base reads.

Raw reads were processed to remove low quality and aligned to the Mus musculus (mm10) using HISAT v2.1.0. The reference genome sequence and annotation data were downloaded from the UCSC table browser (http://genome.ucsc.edu). Transcript assembly and abundance estimation were performed using StringTie v2.1.3b. Differentially expressed genes (DEGs) were measured using the estimate of abundance for each gene in the samples with indicated treatments, considering the six RNA‐seq experiments as biological replicates. Statistical significance of the DEGs was determined using edgeR. Hierarchical clustering analysis also was performed using complete linkage and Euclidean distance as a measure of similarity to display the expression patterns of differentially expressed transcripts which are satisfied with |fold change| ≥ 2 and independent *t*‐test raw *p* < .05. The log2 fold change and *p*‐value were obtained from the comparison of the average for each group plotted as the volcano plot. (*X*‐axis: log2 Fold Change, *Y*‐axis: −log10 *p*‐value). All data analysis and visualization of differentially expressed genes were conducted using R 3.5.1 (www.r-project.org). Raw data can be accessed through the Gene Expression Omnibus (GEO) repository with accession number GSE 186765.

### 2.11 Statistical analysis

Data are presented as mean ± SEM for all data. Comparisons were made by one‐way ANOVA, with significance being defined as *p* < .05 (*), *p* < .01(**), and *p* < .001 (***) using GraphPad Prism 7.01. For multiple comparisons, one‐way ANOVA with Tukey's multiple comparison test was used.

## 3 RESULTS

### 3.1 Ginsenoside Rh2 sensitized doxorubicin in human tumour xenograft mice

**FIGURE 1:**
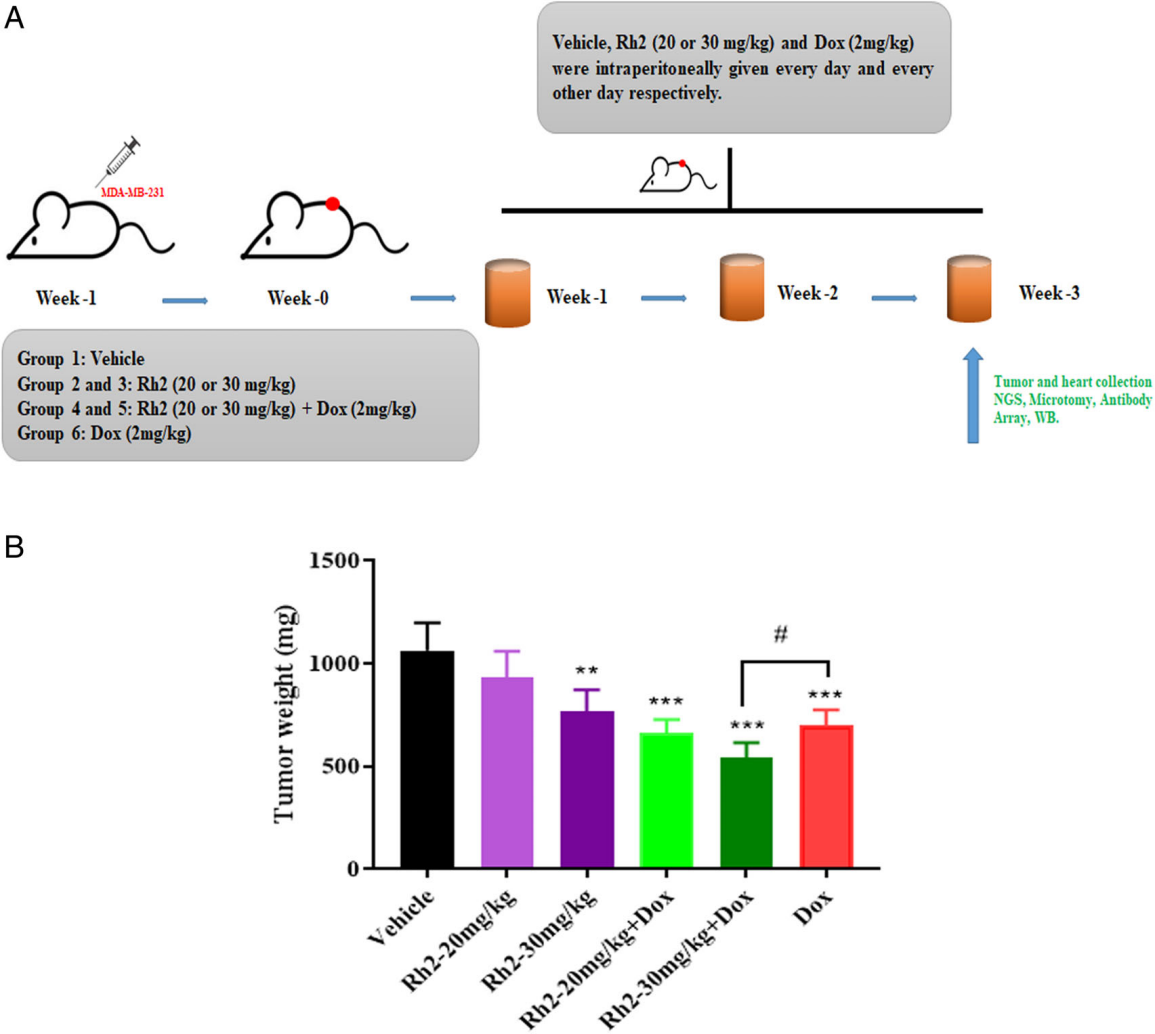
Ginsenoside Rh2 synergistically sensitize the chemotherapeutic toxicity of Doxorubicin in breast cancer xenograft. (A) Schematic for experimental regime. (B) Tumour weight. *p < .05; **p < .01; ***p < .001 versus control; ^#^ *p* < .05; ^##^ *p* < .01; ^###^ *p* < .001 versus Dox

To examine the anticancer effect, breast cancer‐bearing BALB/c nude mice were treated with saline, ginsenoside Rh2 of 20 and 30 mg/kg with or without 2 mg/kg doxorubicin, and doxorubicin alone for 3 weeks starting at age 7 week (Figure 1A). Doxorubicin‐treated mice demonstrated a progressive decrease in tumour weight that became substantial 2 weeks following treatment, while these changes were more remarkable in ginsenoside Rh2 combination groups (Figure 1B).

### 3.2 Ginsenoside Rh2 attenuated doxorubicin‐induced cardiac pathology

**FIGURE 2:**
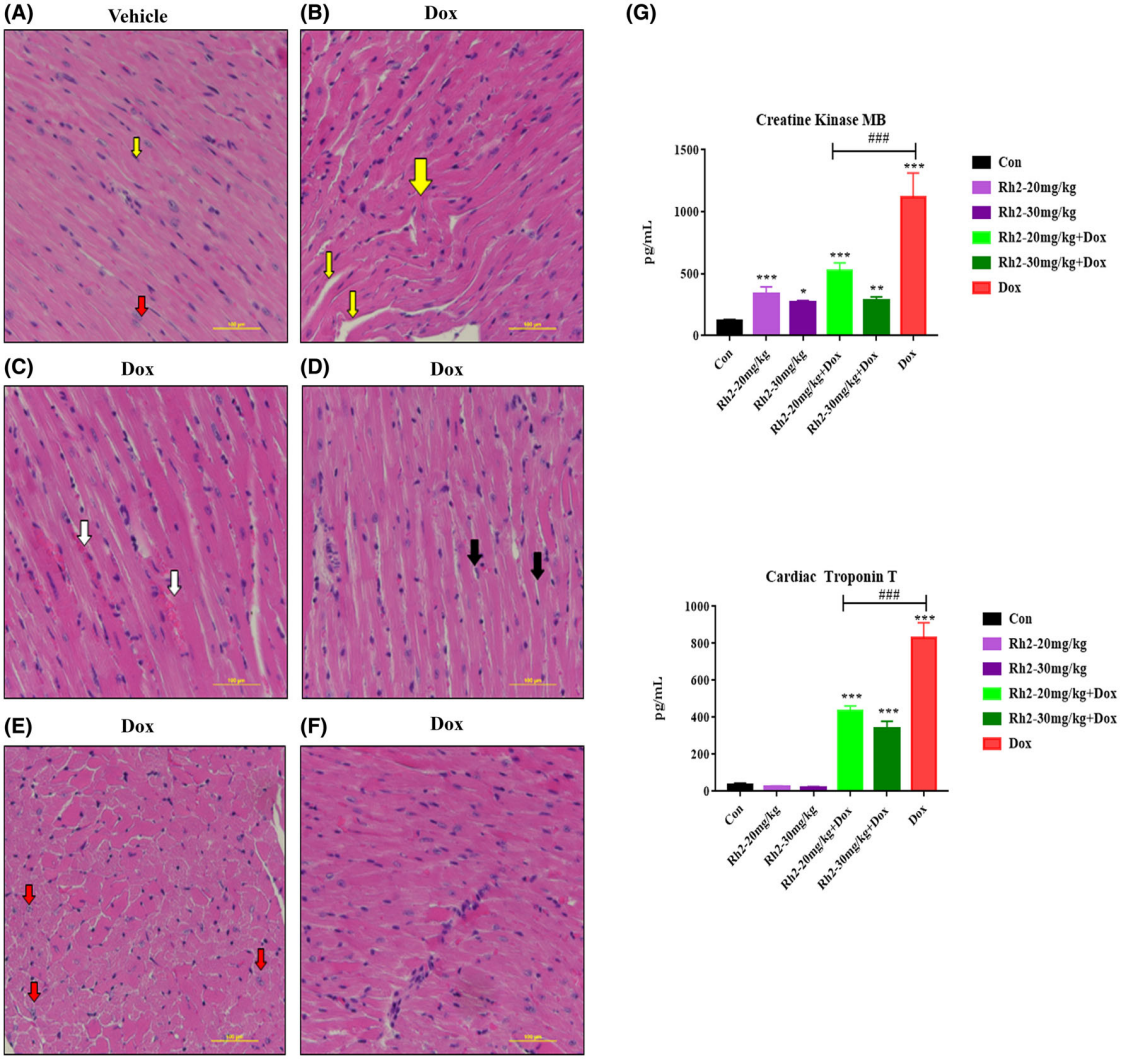
Histological examination of the heart. (A) The normal cardiac muscle fibres are longitudinally settled with oval nuclear and acidophilic striated sarcoplasm (red arrow). Flat fibroblasts constitute the interstitial space (yellow arrow). (B) Disorganization and separation of cardiac fibres with heightened interstitial spaces (yellow arrow). (C) Extravasation of red blood cells leading to interstitial haemorrhage (white arrows). (D) Cardiac damage was characterized by myofiber vacuolation (black arrows). (E) Focal myocardial necrosis into areas of preserved myocardium (red arrows). (F) Mononuclear cellular infiltration. Scale bars = 100 μm. (G) ELISA assays of CK‐MB and Troponin T release in cardiomyocytes. *p < .05; **p < .01; ***p < .001 versus control; ^#^ *p* < .05; ^##^ *p* < .01; ^###^ *p* < .001 versus Dox

Mice without treatment of doxorubicin displayed a normal histological pattern of the myocardium. Cardiomyocytes have cylinder‐shipped fibres with oval central vesicular nuclei. Fibroblast lies between the cardiac fibres with flat nuclei (Figure 2A). H&E staining of the doxorubicin‐treated group demonstrated severe histological changes in the form of disorganization of the fibres with an increase in the interstitial gaps between these fibres (Figure 2B). Extravasation of the red blood cells with interstitial haemorrhage emerged in some restricted areas (Figure 2C). Pathology was dominated by areas of intracytoplasmic vacuolation accompanied by considerable heterogeneity in myofiber diameter, and pathed myocardial necrosis (Figure 2D and E). Cell recruitment was also observed in the interstitial tissue (Figure 2F). Additionally, ELISA assays of CK‐MB and Troponin T released from cardiomyocytes notified the damages induced by dox (Figure 2G). Collectively, the heart of Dox‐treated mice showed severe histological damage with congestion, rippled myocytes, reduction of striated muscle bands, hemorrhagic areas, myocytolysis, and focal necrosis, effects that were markedly mitigated by ginsenoside Rh2 treatment. Interestingly, heart weight did not statistically differ from the vehicle‐treated group with a minor reduction of 6.3%. Thus, our results suggest that ginsenoside Rh2 preserves the cardiac structure by reducing histological damage.

### 3.3 Protection of Rh2 on doxorubicin‐damaged heart exhibited distinct transcriptome profiles

**FIGURE 3:**
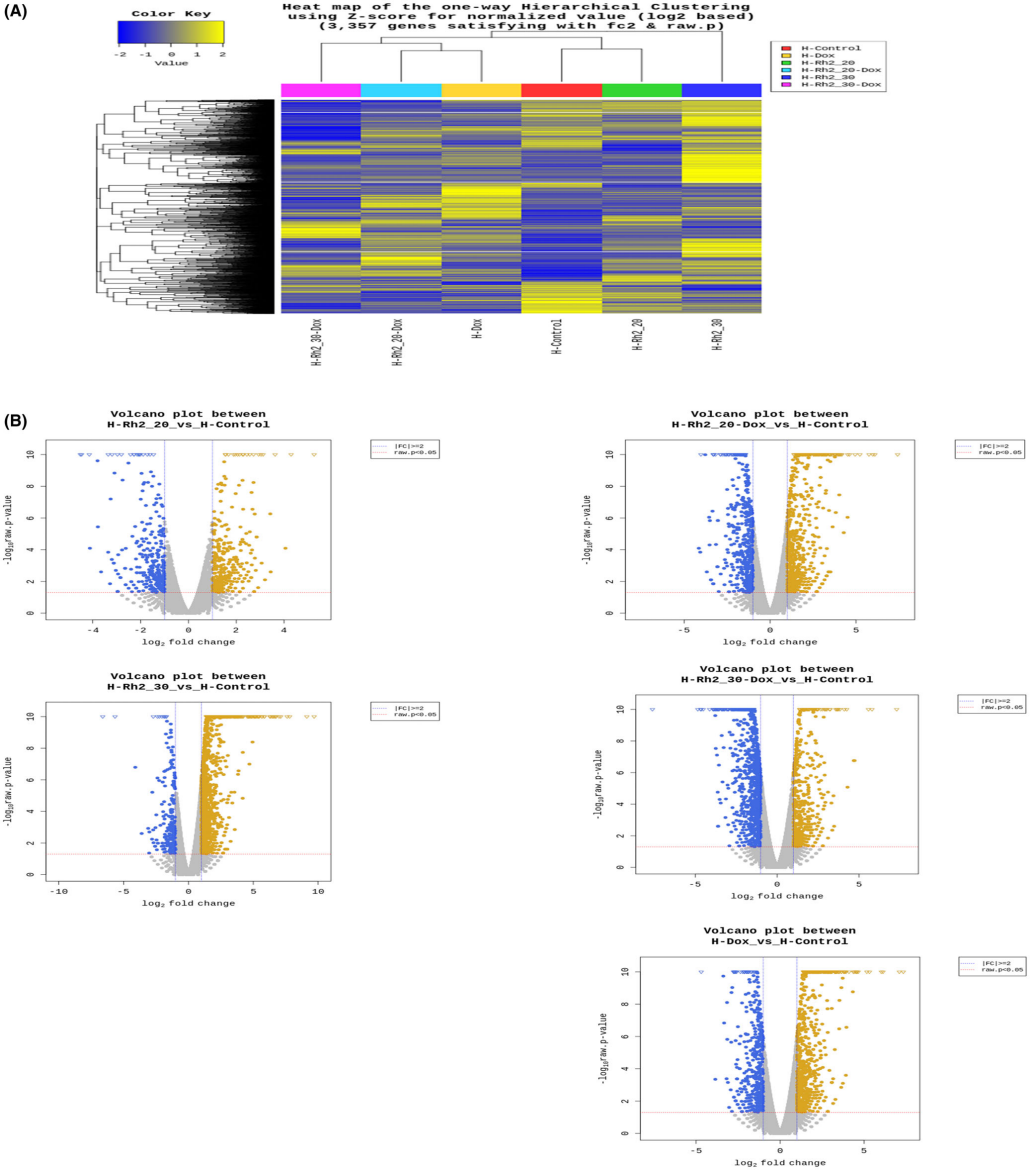
The protective effect of Rh2 on the heart exhibited distinct functional properties and transcriptome profiles. (A) Heatmap of DEGs in six groups: Control, Rh2‐20 mg/kg, Rh2‐30 mg/kg, Rh2‐20 mg/kg + Dox, Rh2‐30 mg/kg + Dox, and Dox‐2 mg/kg. Gene expression is represented as *Z*‐score for the normalized value across rows. (B) The volcano plots of DEGs in the indicated groups

**FIGURE 4:**
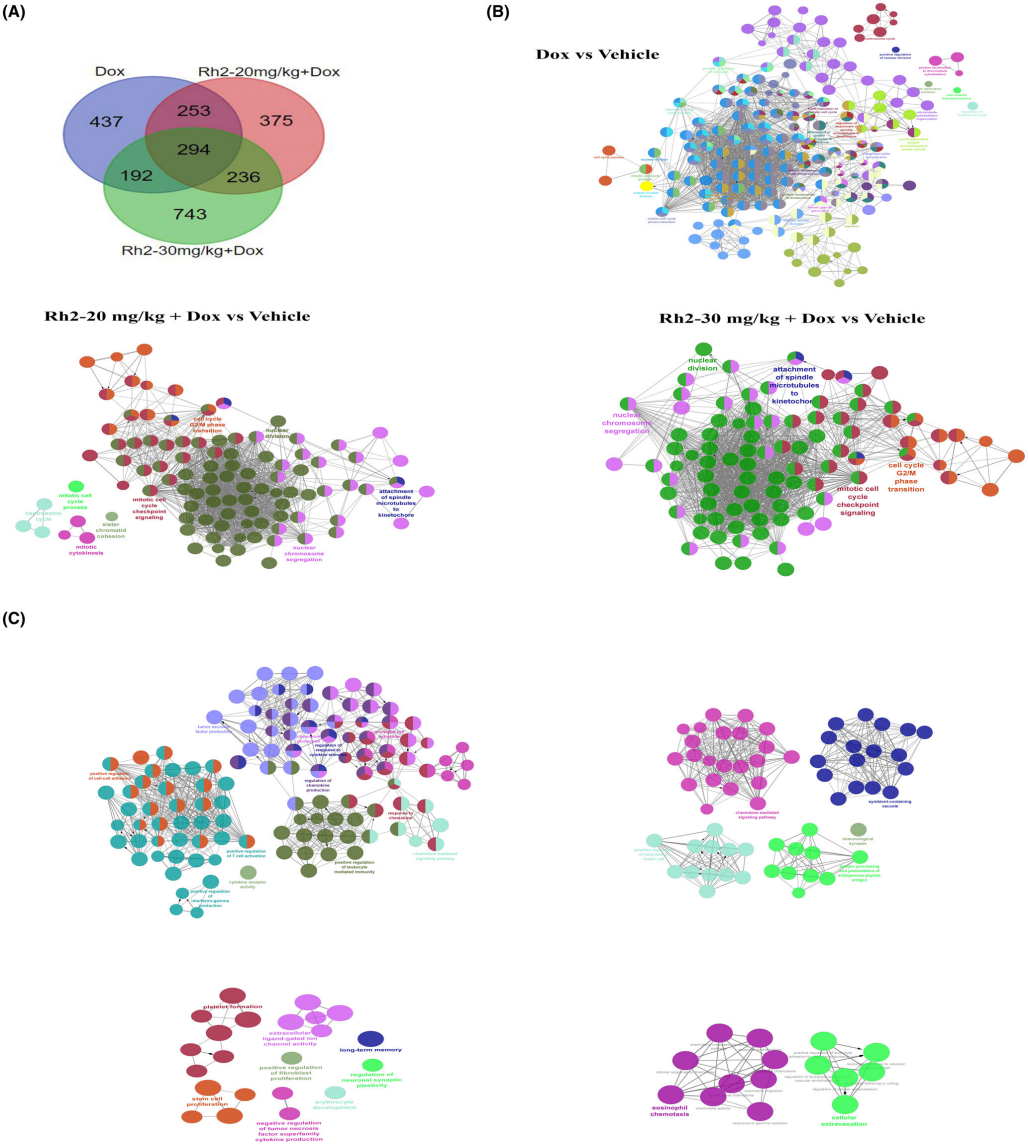
Functional and pathway enrichment analysis of DEGs. (A) Venn diagram of genes common or unique in Dox, Rh2‐20 mg/kg + Dox and Rh2‐30 mg/kg + Dox, as compared to Control group. (B) Network of the enrichment pathways for the common DEGs. (C) Network of the enrichment pathways for the unique DEGs

To further delineate the underlying protective mechanism of Rh2 on the doxorubicin‐challenged heart, we analysed the transcriptomic alterations of the hearts by RNA‐sequencing. A total of 3357 DEGs were identified in hearts from the breast tumour‐bearing mice treated with doxorubicin (combined with or without Rh2) or vehicle group (Figure 3A). Volcano plots indicated DEGs between treatment groups and vehicle groups individually with 1866 upregulated ones and 1491 downregulated ones (Figure 3B). Overall, these identified DEGs can distinguish the chemotherapeutic groups from non‐treated groups. A total of 294 genes were overlapped among Dox group, Rh2‐20 mg/kg + Dox group and Rh2‐30 mg/kg + Dox group (Figure 4A). These commonly expressed genes are predominantly implicated in cell cycle and microtubule attachment (Figure 4B), and unique profiles from the Dox group revealed the positive regulation of inflammation comprising chemokine, cytokine, tumour necrosis factor production, and immune cell activation (Figure 4C). Profile changes were annotated by Gene Ontology (GO) analysis and visualized by Cytoscape software.

### 3.4 Ginsenoside Rh2 alleviated doxorubicin‐induced apoptosis in the heart

**FIGURE 5:**
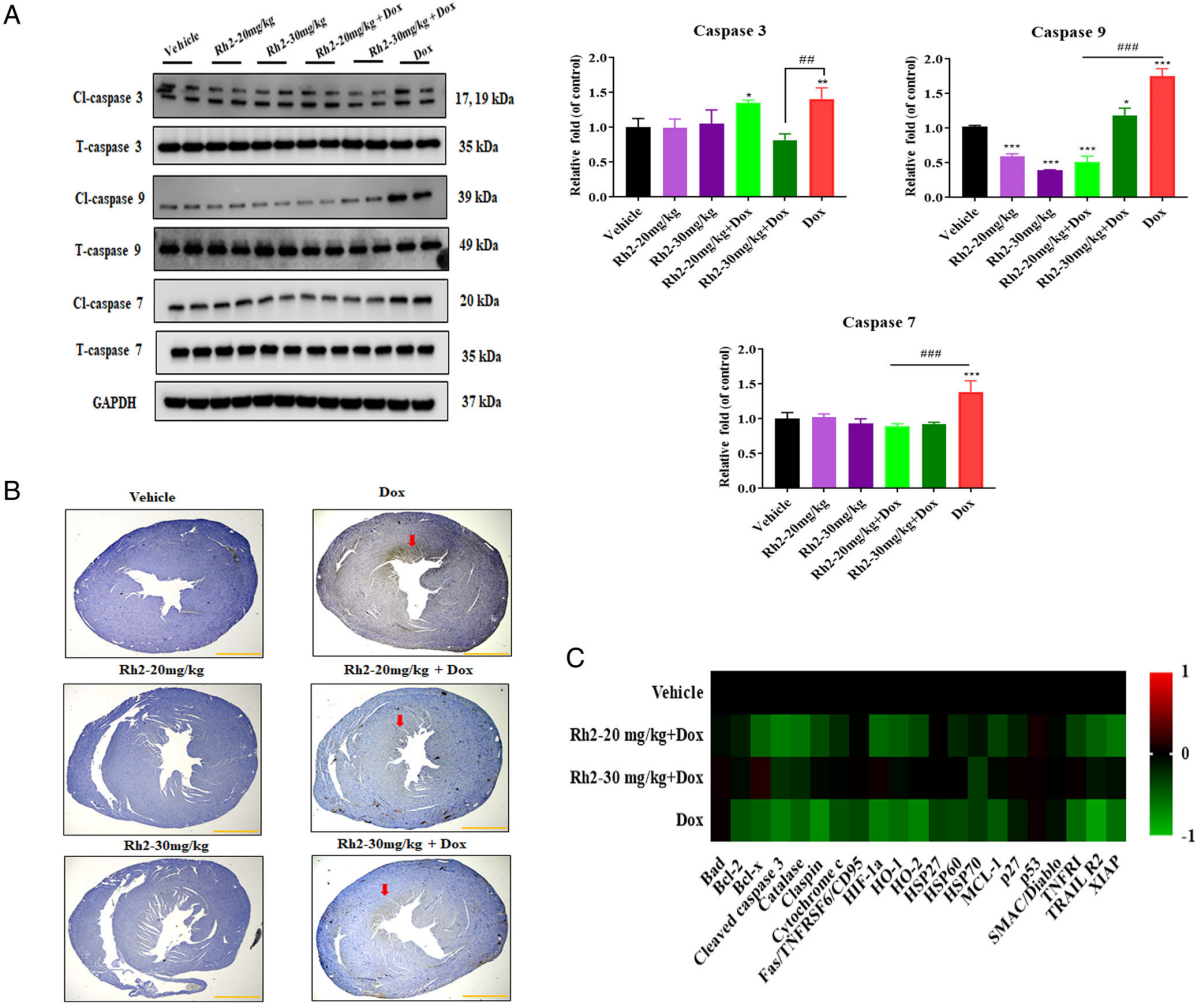
Rh2 alleviated cardiac apoptosis. (A) Extracts from mice heart treated with or without ginsenoside Rh2; Doxorubicin‐challenged mice, treated or not with ginsenoside Rh2, were assayed by western blotting for the indicated apoptosis marker proteins caspase 3, 7, and 9. GAPDH was used as a loading control. (B) Cleaved caspase 3 staining on the doxorubicin‐evoked heart in mice (positive staining mainly located at left ventricle and indicated with red arrows). Scale bars = 100 μm. (C) Heart tissues from mice treated with doxorubicin combined with or without ginsenoside Rh2 or vehicle were analysed by apoptosis antibody arrays. Signals from vehicle‐treated mice were used as a baseline. Colour intensities represent log2‐fold changes from baseline. Signals higher than the baseline are shown in red; signals lower than the baseline are shown in green. Shown is the average of three independent experiments. *p < .05 versus control; ^#^ *p* < .05 versus Dox

To investigate the consequence of cardiac pathology, heart tissues were analysed using western blotting against topical targets. The degree of cardiac apoptosis increased as manifested by increased levels of apoptotic protein markers cleaved caspase 3, 7, 9, and PARP (Figure 5A). Consistent with immunoblot results, positive cleaved caspase 3 stained cells in mice hearts were significantly elevated following doxorubicin treatment; however, 20 and 30 mg/kg ginsenoside Rh2 administration caused a remarkable decrease (Figure 5B). To reveal the other attributive components in doxorubicin‐induced apoptosis, heart tissues were applied to an antibody array with 21 mouse apoptosis‐related proteins. In the doxorubicin‐treated group, of factors detected by arrays and expressed at high levels are pro‐apoptotic cleaved caspase 3, p53, SMAC, and TRAIL R2; low levels are Bcl‐2, Bcl‐x, Catalase, HO‐2, HSP27, HSP60, and XIAP (Figure 5C). We postulated that ginsenoside Rh2 partly rescued the cardiac cells from apoptosis, thereby suppressing the heart damage induced by doxorubicin in breast tumour‐bearing mice.

### 3.5 Ginsenoside Rh2 repressed doxorubicin‐induced heart inflammation

**FIGURE 6:**
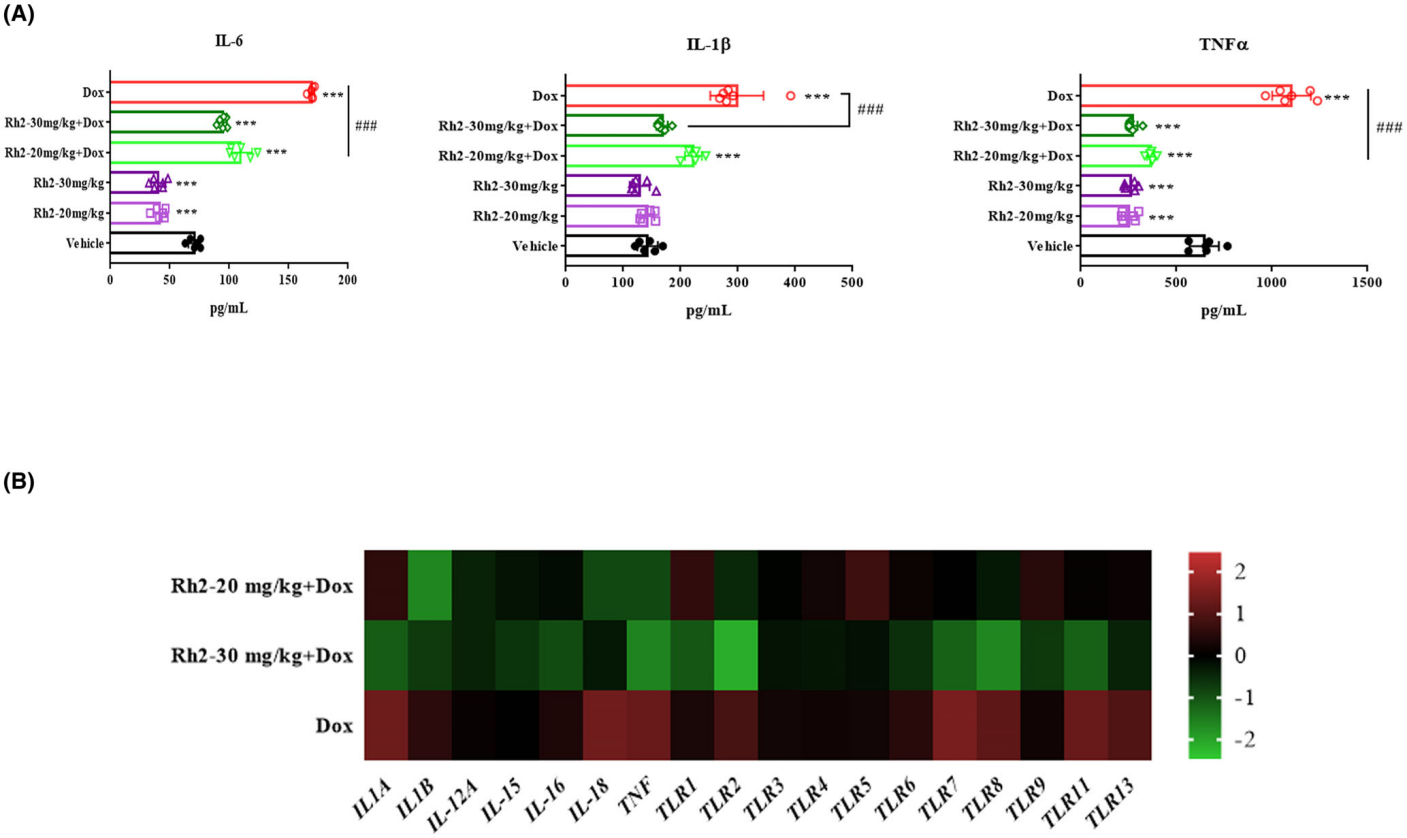
Rh2 suppressed cardiac inflammation. (A) Heart lysates were analysed by ELISA for TNF‐α, IL‐6, and IL‐1β. Quantifications were shown. (B) Fold changes of mRNA levels of adverse remodelling markers. Levels of each protein in the control group were arbitrarily set to zero. Data shown represent a log2‐fold change in expression relative to control. *p < .05 versus control; ^#^ *p* < .05 versus Dox

Doxorubicin induces cardiac necrosis and causal inflammation that exerts sustained deleterious effects on the heart. Thus, we measured the protein levels of TNF‐α, IL‐6, and IL‐1β using ELISA assay. Rh2 significantly lowered the expression of IL‐1β and TNF‐α, and to a lesser extent IL‐6, in doxorubicin‐evoked hearts (Figure 6A). Additionally, doxorubicin remarkably increased the levels of TLR2, TLR6, TLR7, TLR8, TLR11, and TLR 13, as expected. In the presence of Rh2, all the elevated ones were decreased efficiently at both doses. However, TLR1 and TLR5 were increased exclusively in 20 mg/kg of the Rh2 + Dox group. Apart from TLRs, doxorubicin considerably amplified the cytokines including IL‐1A, IL‐18, and TNF. Rh2 at both doses decreased all the transcriptional levels of these cytokines (Figure 6B). Thus, Rh2 suppressed doxorubicin‐induced cardiac damage‐associated sterile inflammation.

### 3.6 Ginsenoside Rh2 mitigated doxorubicin‐induced cardiac fibrosis

**FIGURE 7:**
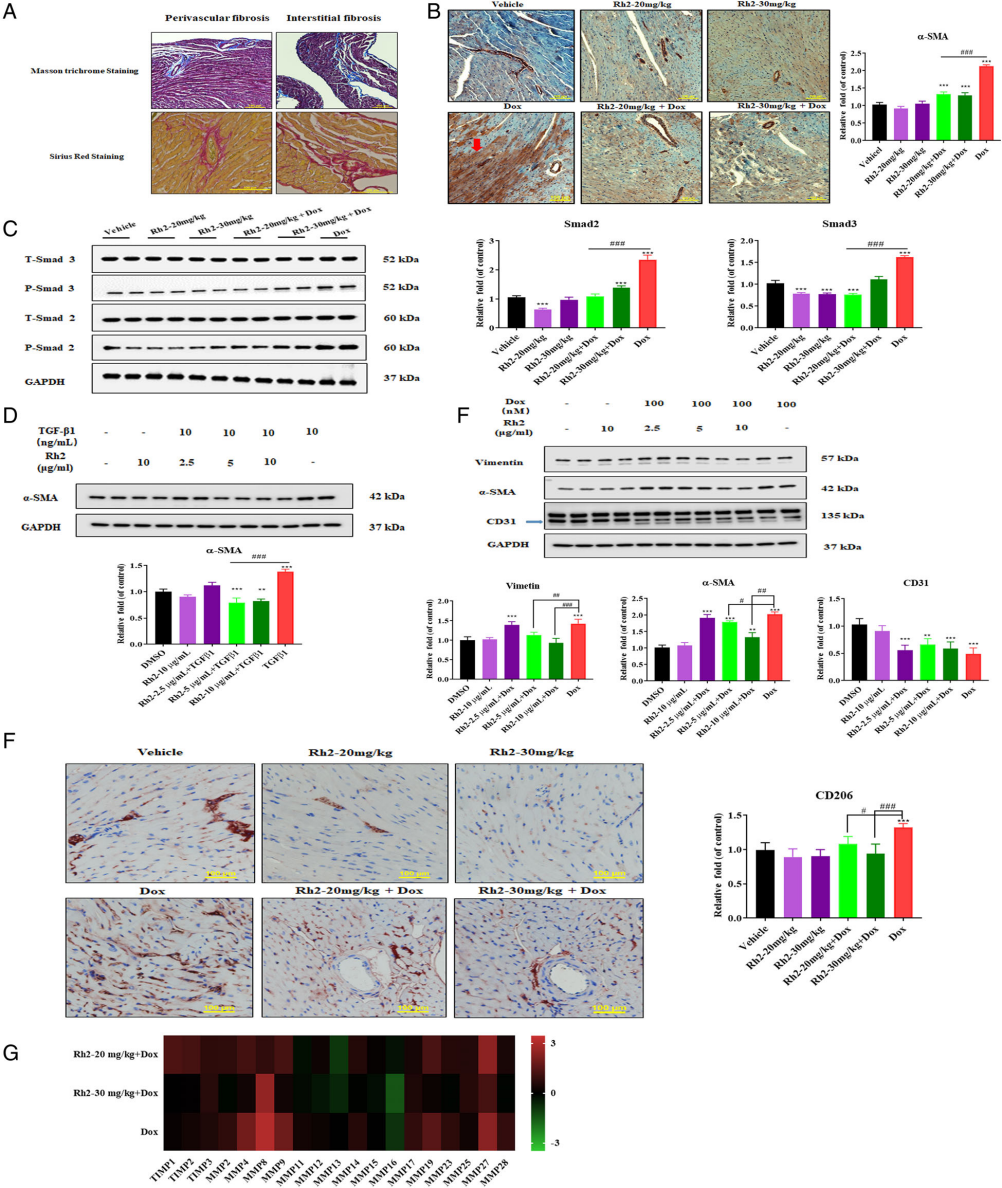
Rh2 Attenuated cardiac fibrosis. (A) Heart sections were stained to identify fibrosis by Masson trichrome and Sirius Red methods. Shown are representative images of interstitial and perivascular fibrosis from doxorubicin‐treated mouse hearts. Scale bars = 100 μm. (B) IHC staining of α‐SMA in doxorubicin‐administrated mouse myocardium. Red arrows indicate replacement fibrosis. Scale bars = 100 μm. (C) Downstream effectors smad2 and smad3 protein levels in heart lysates were analysed using western blot. GAPDH was used as a loading control. (D) Human cardiac fibroblasts were exposed to ginsenoside Rh2 at 2.5, 5, 10 μg/ml for 12 h, then subjected to 10 ng/ml of TGF‐β1 for extra 24 h. Protein extracts were analysed with the indicated antibodies. GAPDH was used as a loading control. (E) HUVECs were treated with 100 nM doxorubicin for 7 days, followed by 2.5, 5, 10 μg/ml of Rh2 exposure for extra 7 days. Cell extracts were incubated with endothelial markers CD31 and myofibroblast markers α‐SMA and vimentin. Beta Actin was used as a loading control. (F) Detection of CD206 expression in the vehicle and doxorubicin‐treated heart sections by immunohistochemistry analysis. Scale bars = 100 μm. (G) Fold changes of mRNA levels of inflammation markers. Levels of each protein in the control group were arbitrarily set to zero. Data shown represent a log2‐fold change in expression relative to control. Signals higher than control were shown in red; signals lower than control were shown in green. *p < .05 versus control; ^#^ *p* < .05 versus Dox

Fibrosis plays an important role in cardiac dysfunction largely caused by collagen, which invades and replaces necrotic or apoptotic myocytes. Given the abovementioned existence of necrosis and apoptosis, we examined the occurrence of fibrosis by Masson trichrome (MT) and Sirius Red (SR) staining. As expected, an abundance of collagen dramatically increased in the heart of doxorubicin‐treated mice, as indicated by obvious interstitial fibrosis and perivascular fibrosis (Figure 7A). More noteworthy, replacement fibrosis was observed in the present model, which was manifested by IHC staining against fibrosis marker protein α‐SMA. Combined treatment with ginsenoside Rh2 partially decreased the level of fibrosis at both doses (Figure 7B).

To elucidate how fibrosis originates in heart tissue, we next evaluated the regulator proteins in TGF‐β1 signalling. In compared to doxorubicin‐alone‐treated mice, elevated activation of proteins smad2 and smad3 were significantly suppressed by the combination of ginsenoside Rh2. Additionally, fibrosis markers α‐SMA and COL1A1 protein expressed to a lesser extent accordingly (Figure 7C). In culture, Rh2 inhibited fibroblast to myofibroblast transition (FMT) when cardiac fibroblasts were exposed to TGF‐β1 for 24 hours, demonstrating that Rh2 targeted TGF‐β signalling (Figure 7D).

As the endothelial–mesenchymal transition (EndMT) process can give rise to activated myofibroblasts involved in many fibrotic diseases, we tested the possibility that Rh2 decreased EndMT in dox‐challenged fibrosis herein. We treated human umbilical vein endothelial cells (HUVEC) with doxorubicin, followed by exposure of various concentrations of Rh2 (2.5, 5, and 10 μg/ml), and measured the marker proteins by western blot analysis. Doxorubicin reduced the levels of CD31 protein, whereas it increased specific cell markers such as α‐SMA and vimentin. Intriguingly, Rh2 despite eliciting little change in endothelial markers, significantly reduced the α‐SMA and vimentin protein levels in doxorubicin‐exposed cells (Figure 7E).

Since necrotic myocytes release endogenous damage‐associated molecular patterns (DAMPs), which activate resident macrophages to release pro‐inflammatory cytokines that trigger tissue fibrosis, we evaluated CD206 positive cells in the heart section by immunohistochemistry analysis. In the presence of Rh2 and doxorubicin, CD206 positive cells were significantly reduced. In contrast, doxorubicin alone group, CD206 positive cells were greatly increased, indicating vigorous fibrosis induction (Figure 7F).

We sorted the fold changes of the matrix metalloproteinases (MMPs) and their inhibitors (TIMPs) in the heart tissues that are closely related to cardiac remodelling following damage (Figure 7G). Doxorubicin increased MMPs levels of MMP2, MMP4, MMP8, MMP9, MMP14, MMP17, MMP19, MMP23, MMP27, and MMP 28. In the presence of 30 mg/kg Rh2, all the MMP detected were suppressed except MMP8 and only TIMP3 was slightly elevated. Additionally, Rh2 at 20 mg/kg suppressed MMP8, MMP17, and MMP28, while increasing TIMP1, TIMP2, and TIMP3. Interestingly, MMP 13 and MMP16 were reduced by both Rh2 groups and Dox and Rh2 30 mg/kg groups.

Strikingly, doxorubicin‐challenged heart developed severe fibrosis. In contrast, ginsenoside Rh2 moderately ameliorated this stress.

### 3.7 Ginsenoside Rh2‐induced premature senescence of myofibroblasts

**FIGURE 8:**
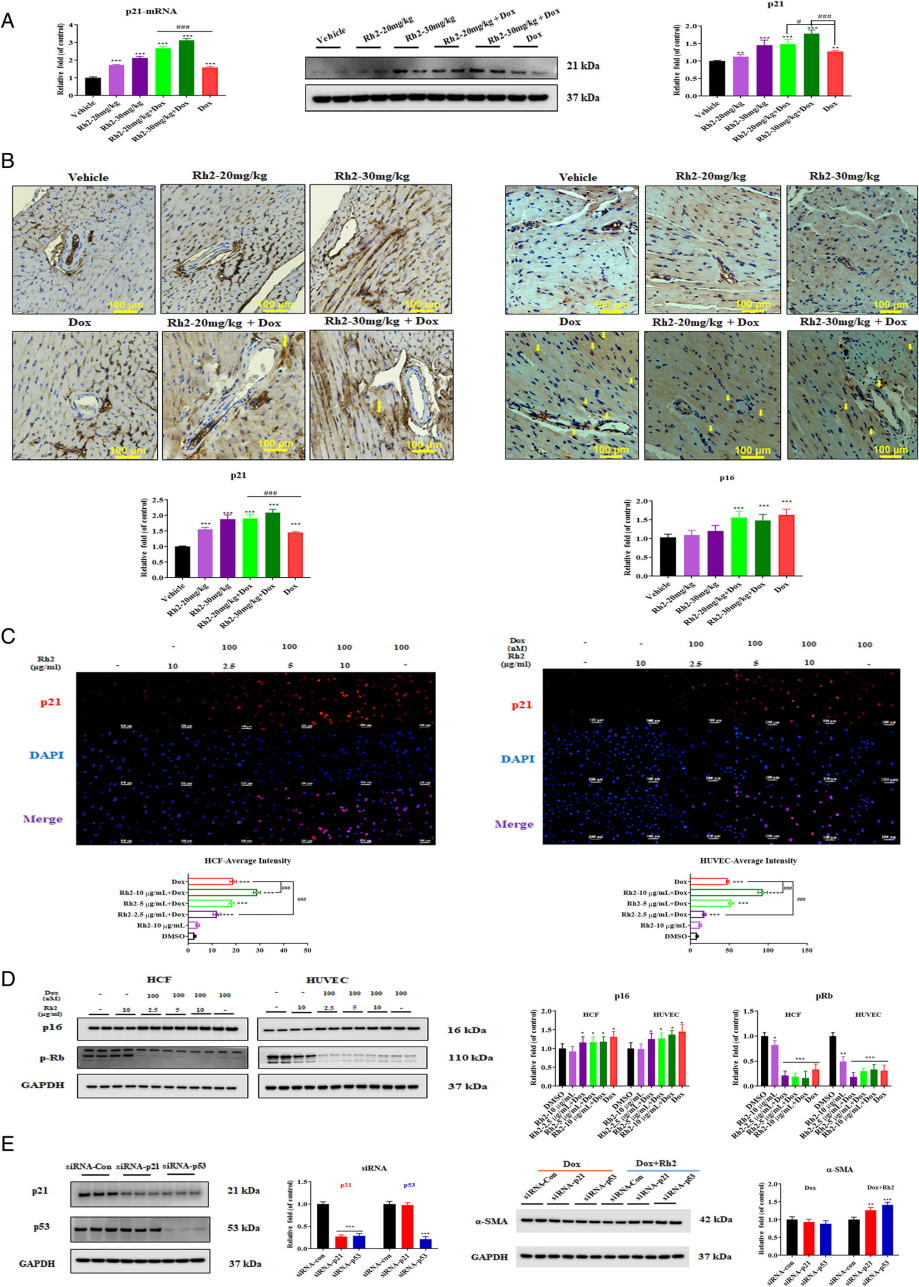
Rh2 specifically induced senescence in cardiac fibroblasts differentiated myofibroblasts. (A) Heart tissues were analysed with p21 at both mRNA and protein levels. Quantifications were shown. GAPDH was used as a control. (B) Representative sections of hearts stained with p21 and p16 and quantifications. Positive cells were indicated by yellow arrows. Scale bars = 100 μm. (C) HCF and HUVEC cells were treated with 100 nM doxorubicin for 7 days, followed by treatment with Rh2 or DMSO for 7 days. Cells were then fixed, washed, and probed with the p21 antibody. The expression level of p21 was determined by fluorescence microscopy (one representative of three independent experiments is shown). Scale bars = 100 μm. (D) Protein levels of p16 and phosphorylated Rb were analysed by western blot. GAPDH was used as a loading control. (E) p21 or p53 silenced Dox‐treated HCF cells were analysed for α‐SMA by western blot. Cells were treated with or without 10 μg/ml of Rh2 for 48 h and then transfected with siRNAs for extra 48 h; quantifications of siRNA efficiency of p21 and p53 were shown. GAPDH was used as a loading control. *p < .05 versus control; ^#^ *p* < .05 versus Dox

As premature senescence of myofibroblast may prevent proliferation and trigger matrix degradation, Rh2 might be a possible inducer of senescence in myofibroblast. After combination treatment with Rh2 for 3 weeks, mice showed increased levels of both mRNA and protein of p21, a senescence marker, in the heart tissues than the doxorubicin individual group (Figure 8A). Increased levels of p21 and p16 were detected by immunohistochemistry in heart sections. A higher level of p21 in myofibroblast was explicitly observed in Rh2 combination groups, and a significant elevation of p16 was confirmed in myocardiocytes of Dox‐treated mouse hearts. (Figure 8B). To identify the senescent cells, we further assessed the protein levels of senescence markers using immunocytochemistry (Figure 8C) and western blot (Figure 8D) in Dox‐exposed human cardiac fibroblasts (HCF) and HUVEC cells. In both cell lines tested, Rh2 significantly increased the expression level of p21 compared with the doxorubicin alone group, while little changes were observed in p16/pRb signalling. To evaluate the biological relevance of premature senescence in myofibroblast differentiation, we investigated the cardiac fibroblasts with p21 or p53 knockdown by siRNA. Results showed that p21 or p53 knockdown significantly reduced the suppression of Rh2 on the expression of myofibroblast marker α‐SMA (Figure 8E). Thus, Rh2 may attenuate excessive fibrosis following Dox treatment by specifically enhancing premature senescence in cardiac fibroblast differentiated myofibroblasts.

## 4 DISCUSSION

**FIGURE 9:**
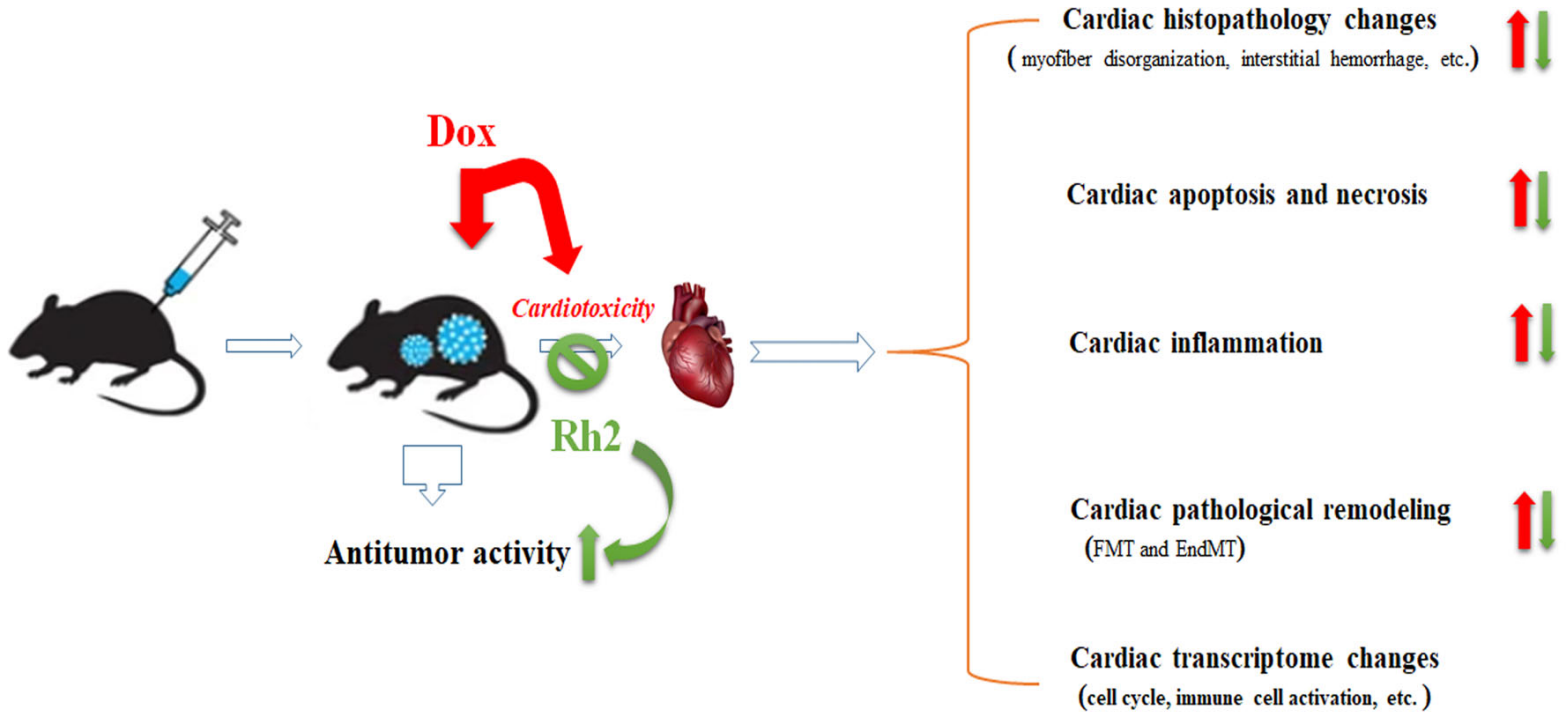
Schematic representation of protection by Rh2 against Dox‐mediated cardiotoxicity in breast tumour‐bearing mice

Dox, a prototype agent of anthracycline antibiotics, has been widely applied but limited to its acute and chronic cardiotoxicity.^18^ Indeed, natural compounds might be effective for the synergistic action of Dox while protecting against cardiac damage.^19^ In the present study, we interrogated ginsenoside Rh2 as a viable supplement for Dox application in cancer treatment. Our findings herein demonstrated that ginsenoside Rh2 ameliorated Dox‐induced cardiotoxicity and simultaneously enhanced its anticancer activity in a breast cancer‐bearing mouse model (Figure 9). These results indicated that Rh2 afforded its protection by reducing cardiac apoptosis, inflammation, and fibrosis evidenced by histological and pathological observation, immunoblotting analysis, and RNA‐sequencing analysis. We identified the transcriptome changes of hearts among groups by RNA‐sequence analysis that provided several readouts: (1) Dox treatment predominantly targets the cell cycle and attachment of microtubules in heart, (2) Dox specifically exhibits a hyperactive immune response including tumour necrosis, chemokine, and interferon‐gamma production, response to cytokine and chemokine, and T‐cell activation. (3) Dox pointedly targets fibroblast and stem cell proliferation. (4) Rh2 markedly targets the hyperactivation of cytokines and immune cells.

We examined the histological pattern of the myocardium. Dox‐induced pathological changes with dominated areas of apoptosis and necrosis, which are the most plausible intracellular signalling mechanisms underlying cardiotoxicity.^20^ Generally, Dox promotes apoptosis by regulating proapoptotic proteins and death receptors. Our apoptosis array data validated the higher expression of proapoptotic proteins and TRAIL‐R2 and further showed suppressed levels of Catalase, HO‐2, HSP27, HSP60, and XIAP, suggesting activation of both intrinsic and extrinsic pathways. These proapoptotic factors were remarkably reduced by Rh2 treatment, notably with the 30 mg/kg group. Moreover, pathed myocardial necrosis with intracytoplasmic vacuolation was observed, accompanied by proinflammatory cytokine release and inflammatory cell infiltration. Studies have formerly indicated that Dox induces mitochondrial hyper‐activation, ROS generation, DNA damage, and consequent necrosis.^21^ Furthermore, necrotic myocardial cells release DAMPs that can sufficiently provoke myocardial inflammation.^22^ Our results revealed strong Toll‐like Receptor (TLR) activations after the Dox challenge. This might be the mechanism behind necrosis‐associated cardiomyopathy since TLRs could recognize various DAMPs released from necrotic cardiomyocytes.^23^ Correspondingly, inflammatory factors such as IL‐6, IL‐1β, and TNFα were significantly elevated at both mRNA and protein levels after Dox treatment. However, combination with ginsenoside Rh2 significantly reduced the levels of apoptosis‐related proteins, inflammatory factors, and necrosis, leading to attenuated cardiac damage. Additionally, we can conclude the possible contribution of tumour‐derived systemic inflammation to the higher levels of cytokine release in Dox‐free control groups.

Histological observations of cardiac apoptosis and necrosis may well explain the latent cardiomyopathy with severe dysfunctions in patients treated with Dox. Support for this hypothesis comes from the well‐documented cardiac fibrosis that is a common cause of mortality.^24^, ^25^ Sterile inflammation contributes to the heart injury by releasing pro‐inflammatory cytokines and TGF‐β to initiate a fibrotic response. Our present study revealed remarkably increased CD206 positive M2‐macrophage in Dox‐challenged mouse heart. Consequently, TGF‐β/Smad signalling pathways were robustly triggered by the contribution of these inflammatory cells. The results implicated the macrophage in Dox‐involved cardiac fibrosis, which is in agreement with previous research that claims innate immunity effector cells as the drivers of cardiac fibrosis.^26^ Myofibroblast activation is generally derived from EndMT and FMT pathways via various fibrotic mediators, and in cultures, we found that Dox treatment was able to activate both EndMT and FMT pathways. Intriguingly, with the combination of Rh2, Dox‐related excessive macrophage differentiation and EndMT and FMT activation were partly attenuated, which may represent the essential mechanism underlying the anti‐fibrotic effect. Furthermore, by regulating extracellular matrix (ECM) degradation, MMPs play a vital role in the development of fibrosis, and some are closely related to cardiomyopathy and heart failure.^27^, ^28^ The combination of Rh2 and Dox proportionally suppressed the levels of selected MMPs compared with the single Dox treated group. Nonetheless, based on the present results, we speculated that Rh2 also regulated cardiac fibrosis with MMPs.

Cell cycle arrest, a common consequence of Dox treatment, is one of the drivers of cellular senescence. Senescent cells are believed to contribute to a wealth of diseases, especially aging‐related pathologies. We observed the induction of senescence in cardiac fibroblasts and blood vessel endothelial cells as well as cardiomyocytes in response to Dox. Moreover, in cardiac fibroblasts, elevated senescence markers are accompanied by increased levels of α‐SMA and vimentin, suggesting senescence occurs in myofibroblast differentiation triggered by Dox, and Rh2 specifically enhanced the premature senescence identified by ICC and IHC analyses. This effect may partially explain the antifibrotic mechanism of Rh2 since it is in line with a previous report claiming that premature senescence in myofibroblast is a potential therapeutic target in myocardial fibrosis.^29^ Furthermore, our results also demonstrate that Rh2 suppresses the expression of myofibroblast marker α‐SMA via senescence, which is manifested by knockdown of p21 or p53 in cardiac fibroblasts. Intriguingly, after exposing Dox‐provoked blood vessel endothelial cells to Rh2, we observed a decrease in myofibroblast markers α‐SMA, indicating that Rh2 is likely to reverse the established myofibroblast differentiation and further ameliorate the EndMT‐Fibrosis process. Although previous studies demonstrated that it was able to reduce the risk of fibrosis via reversible modulation of myofibroblast differentiation,^30^, ^31^ further experiments are warranted to explain the putative mechanism underlying Rh2. Senescence in cardiomyocytes involves a decreased replicative capacity and impairment of reparative and regenerative ability,^32^ which facilitates Dox‐induced cardiotoxicity. Indeed, we confirmed senescence in cardiomyocytes by increased protein levels of p16 and p21 after the Dox challenge, while treatment of Rh2 did not statistically affect senescence.

Although we demonstrated Rh2 to protect the heart from apoptosis and necrosis, inflammatory response, and fibrosis, explicit causal interactions among these factors regulated by Rh2 were not well‐described in the present study. Herein, the treatment course lasted for 3 weeks, and acute cellular senescence was triggered. Generally, transient senescence has an important role in addressing the function of heart development and regeneration, whereas the progressive accumulation of senescent cells is implicated in the heart during aging and closely related to the decline in heart function at a late age. Especially, how Rh2 regulates these senescent cells in long‐term or aged models is not clear. Additionally, in the current study, we inferred that lower bioavailability may be attributed to moderate protection. Therefore, experimental datasets are needed to explain the cause and effect relationship and efficient drug delivery in a future study.

Collectively, apart from direct anti‐apoptosis and necrosis, and inflammation, we postulated that Rh2 elicited weakened pathological remodelling to protect the heart from Dox‐induced cardiac injury during cancer treatment. In particular, Rh2, a readily available natural compound, selectively targets myofibroblasts by senescence and reverses established myofibroblast differentiation, which may provide a novel supplement for the Dox regimen.

## AUTHOR CONTRIBUTIONS

Jingang Hou, Yejin Yun and Sunchang Kim designed the work. Jingang Hou collected data and wrote the manuscript. Changhao Cui contributed ginsenoside preparation. All the authors approved this work.

## CONFLICT OF INTEREST

We declare no conflict of interest.

## Data Availability

Data available on request from the authors.
